# Improving Cycle Life and Capacity Retention in PVMPO‖Li Dual‐Ion Lithium‐Organic Batteries Using an EC‐Free and FEC Additive Containing Electrolyte

**DOI:** 10.1002/smtd.202501766

**Published:** 2026-01-14

**Authors:** Sathiya Priya Panjalingam, Somayeh Ahadi, Jakob Michael Hesper, Uta Rodehorst, Sascha Nowak, Birgit Esser, Martin Winter, Peter Bieker

**Affiliations:** ^1^ MEET Battery Research Center Institute of Physical Chemistry University of Münster Corrensstr. 46 48149 Münster Germany; ^2^ International Graduate School for Battery Chemistry Characterization Analysis Recycling, and Application (BACCARA) Corrensstr. 40 48149 Münster Germany; ^3^ Institute of Organic Chemistry II and Advanced Materials Ulm University Albert‐Einstein‐Allee 11 89081 Ulm Germany; ^4^ Helmholtz Institute Münster (HI MS) IMD‐4 Forschungszentrum Jülich Gmbh University of Münster Corrensstr. 46 48149 Münster Germany

**Keywords:** dual‐ion battery, EC‐free electrolyte, FEC, lithium‐organic battery, PVMPO

## Abstract

Electrolytes critically influence the electrochemical performance and cycle life of lithium ion batteries (LIBs). This holds especially for organic redox polymer‐based batteries, such as those employing poly(3‐vinyl‐*N*‐methylphenoxazine) (PVMPO), where solubility limits performance in conventional ethylene carbonate (EC)/ dimethyl carbonate (DMC)‐based electrolytes. Reducing EC content has shown solubility suppression when using ethyl methyl carbonate (EMC) as a co‐solvent, however, capacity fading persists due to PVMPO electrode degradation. To address this degradation, this study explores the use of EC‐free electrolytes, with and without fluoroethylene carbonate (FEC). Electrochemical investigations, UltraViolet/Visible (UV/Vis) spectroscopy, *post‐*cycling Scanning Electron Microscopy (SEM), Energy Dispersive X‐ray Spectroscopy (EDS) mapping, and X‐ray Photoelectron Spectroscopy (XPS) analyses are employed to evaluate solubility, interfacial properties, and electrode integrity. The EC‐free electrolyte system with FEC retains 95 mAh g^‒1^, while that without FEC retains 86 mAh g^‒1^, outperforming the 76 mAh g^‒1^ observed in EC‐based systems after 500 cycles at 1C. FEC containing electrolyte systems display reduced interfacial resistance, fewer surface cracks, and minimal electrode degradation. These findings demonstrate that EC‐free electrolytes, particularly with FEC, effectively suppress electrode degradation and enhance the cycle life of organic LIBs.

## Graphical abstract

The major bottleneck of PVMPO‐based positive electrodes is electrode degradation during extended cycling with EC‐containing electrolytes. This issue is mitigated by using EC‐free electrolytes with FEC additives, which exhibit reduced interfacial resistance, fewer surface cracks, improved capacity retention, and suppressed electrode degradation, thereby enhancing cycling stability.

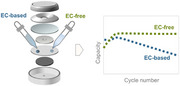

## 1 Introduction

The performance and cycle life of lithium ion batteries (LIBs) are strongly influenced by the composition of the electrolyte. Traditional electrolytes used in conventional LIBs primarily rely to a main part on ethylene carbonate (EC) due to its high dielectric constant and low viscosity, which together enable better ionic conductivity and promote the formation of an effective solid electrolyte interphase (SEI) on graphite negative electrodes.^[^ ^1^, ^2^, ^3^ ^]^ Electrolytes similar to those used in conventional LIBs have also been studied for use with organic electrode materials. In these systems, the electrochemical performance can be strongly affected by the choice of solvent, salt, electrolyte concentration, and additives.^[^ ^4^ ^]^ A deeper understanding of the relationship between solvent composition and organic polymer solubility is therefore essential.

One of the major drawbacks of selected organic electrode materials, particularly small organic compounds such as quinone derivatives and low molecular weight polymers, is their tendency to dissolve in the liquid electrolyte.^[^ ^4^ ^]^ This dissolution not only adversely affects long‐term cycling stability but can also lead to capacity loss due to migration of the dissolved species to the counter electrode, further reducing performance. Although multiple strategies have been explored to mitigate this issue (e.g., structural modification or encapsulation of active materials),^[^ ^5^, ^6^, ^7^, ^8^, ^9^ ^]^ electrolyte optimization remains one of the most effective solutions.^[^ ^10^ ^]^ Unlike other approaches, it does not require introducing inactive components or additional synthesis steps.

Previous reports have demonstrated that lowering the EC content can reduce the polymer solubility. For instance, Perner et al. reported that the redox polymer poly(3‐vinyl‐*N*‐methylphenothiazine) (PVMPT) retained its full theoretical capacity using 1.0 m LiPF_6_ in 3:7 EC:EMC.^[^ ^10^ ^]^ A similar pattern was observed with poly(3‐vinyl‐*N*‐methylphenoxazine) (PVMPO), which has a theoretical specific capacity of 120 mAh g^‒1^ based on a one‐electron redox process and a discharge potential of 3.52 V vs. Li|Li^+^. However, it also exhibited solubility issues when used with 1.0 m LiPF_6_ in 1:1 EC:DMC.^[^ ^11^ ^]^ This issue was mitigated by switching to 1.0 m LiPF_6_ in 3:7 EC:EMC, which led to improved dissolution control and 79% capacity retention after 500 cycles at 1C rate. It also demonstrated good rate capability, retaining 82% of its capacity when cycled at 100C. Despite these improvements, gradual capacity fading was still observed due to PVMPO electrode degradation and deposition on the lithium counter electrode due to undesirable side reactions, which remain as a critical bottleneck.^[^ ^12^ ^]^

This degradation mirrors the “electrode–electrolyte crosstalk,” observed in inorganic systems, i.e., transition metal dissolution from the positive electrode and subsequent deposition on the negative electrode, resulted in rollover failure. The elimination of EC from the standard 1.0 m LiPF_6_ in 3:7 EC:EMC prevented this failure in the high voltage nickel‐rich positive electrodes.^[^ ^13^, ^14^, ^15^ ^]^ Similarly, the addition of additives such as vinylene carbonate (VC) and fluoroethylene carbonate (FEC) have proven effective to stabilize the interfaces in conventional systems.^[^ ^16^, ^17^ ^]^ However, the role of EC‐free electrolytes for organic polymers has not yet been systematically studied.

Building on the degradation observed in PVMPO electrodes employing EC‐containing electrolytes, this study systematically investigates the electrochemical performance of PVMPO‖Li dual‐ion cells using an EC‐free electrolyte, both with and without FEC additive. This study demonstrates that the complete elimination of EC not only suppresses the polymer dissolution but also mitigates electrode degradation more effectively than partially reducing EC. Furthermore, the FEC addition improves the interfacial stability, which resulted in improved accumulated discharge capacity and long‐term capacity retention. Overall, this work highlights that eliminating EC entirely along with the addition of additives provides a viable strategy to suppress side reactions and further proves how a minor change can have a huge influence in improving the performance of redox polymers.

## 2 Results and Discussion

### 2.1 Investigations into the Dissolution Behavior of the Electrolytes

**Figure 1:**
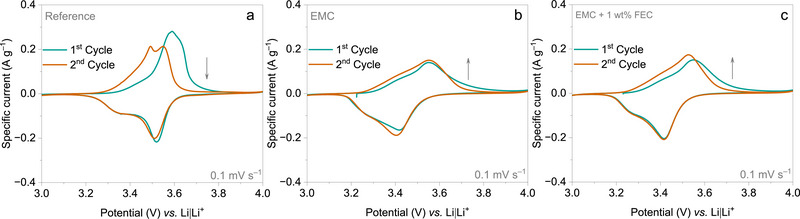
CVs of PVMPO composite electrodes at 0.1 mV s^‒1^. a) Reference b) EMC c) EMC + 1 wt.% FEC electrolytes. The arrow‐indicator shows the trend in current values.

Cyclic voltammetry (CV) is used to assess the solubility of the oxidized polymer in the electrolyte by analyzing the current response during the initial cycles. **Figure** **1**a–c presents the cyclic voltammograms (CVs) of the first two cycles recorded at a scan rate of 0.1 mV s^‒1^ using three different electrolytes. The CVs of the PVMPO composite electrode exhibit reversible redox behavior across all electrolytes, indicating good reversibility.

With the reference electrolyte, the first oxidation peak appears at 3.59 V vs. Li|Li^+^, followed by a reduction between 3.29 and 3.52 V vs. Li|Li^+^. In the second cycle, redox peaks are observed within a similar range (3.29–3.51 V vs. Li|Li^+^). For the EMC electrolyte, the first oxidation occurs at 3.55 V vs. Li|Li^+^ and the corresponding reduction at 3.42 V vs. Li|Li^+^. The second cycle maintains similar behavior, with oxidation at 3.55 V vs. Li|Li^+^and reduction at 3.40 V vs. Li|Li^+^. With the EMC + 1 wt.% FEC electrolyte, the first cycle oxidation and reduction occur at 3.55 V vs. Li|Li^+^ and 3.41 V vs. Li|Li^+^, respectively, while the second cycle shows oxidation at 3.52 V vs. Li|Li^+^ and reduction at 3.42 V vs. Li|Li^+^.

Notably, the first oxidation in the reference electrolyte exhibits a higher current, suggesting enhanced ionic/electronic conductivity and structural reorganization of the polymer due to anion insertion for charge compensation. In contrast, a lower current is observed during the first oxidation in EMC and EMC + 1 wt.% FEC electrolytes. These lower current values can be correlated to the dissolution of the oxidized species, as current values are often linked to the solubility of the active material.^[^ ^10^ ^]^ Interestingly, the second cycle in these electrolytes displays higher oxidation and reduction currents than the first, contradicting the expected dissolution behavior and suggesting a potentially different charge–discharge mechanism.

**Figure 2:**
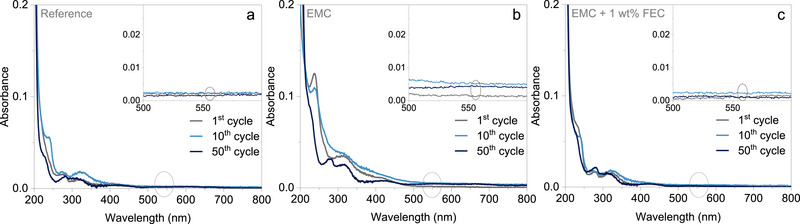
UV/Vis spectra of the three different electrolytes, extracted after the 1^st^, 10^th^ and 50^th^ cycles from the separators a) Reference, b) EMC and c)EMC + 1 wt.% FEC. Insets show a magnified view of the wavelength range 500–600 nm.

To further evaluate whether the dissolution of the oxidized state of the polymer contributes to the observed CV behavior, UV/Vis‐spectroscopic analysis was conducted on electrolytes extracted from the separators after the 1^st^, 10^th^, and 50^th^ cycles. **Figure** **2** presents the UV/Vis spectra for the three different electrolyte samples taken at these respective cycles, with the insets showing a magnified view. Across all three systems, no evidence of dissolution is detected, as indicated by the absence of an absorption band ≈540 nm—the characteristic signal of the oxidized state of PVMPO. This region is highlighted with a grey ellipse in Figure 2 and in the corresponding insets.

**Figure 3:**
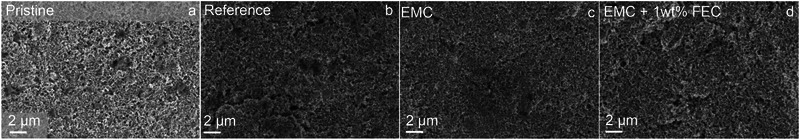
SEM micrographs of PVMPO composite electrodes. a) Pristine electrode before cycling and after the 1^st^ cycle in (b) Reference c) EMC and d) EMC+ 1 wt.% FEC electrolytes.

These results highlight that the observed decreased initial current for the EC‐free systems is not caused by dissolution. This interpretation is further supported by *post*‐cycling SEM measurements, which compare the pristine electrode morphology with cycled samples. **Figure** **3** shows ex situ SEM micrographs of the PVMPO composite electrodes after the first cycle, compared to the pristine electrodes. As anticipated, the SEM images reveal no evidence of dissolution or deposition of the oxidized species, with the morphology of the electrodes after the 1^st^ cycle remaining largely unchanged from that of the pristine samples. This confirms the effective suppression of dissolution and demonstrates good reversibility to the neutral state. Figure S1 (Supporting Information) presents ex situ SEM micrographs of the PVMPO composite electrodes after the 10^th^ and 50^th^ cycles using three different electrolytes. The observed electrode morphologies remain similar to those seen after the 1^st^ cycle.

It is confirmed that the reference electrolyte inhibits dissolution. This is attributed to its lower EC concentration, which facilitates salt dissolution and is associated with polymer dissolution, as well as higher concentration and bulkier nature of the EMC group, which reduces polymer‐solvent interactions and thereby collectively suppressing the solubility.^[^ ^10^ ^]^ A similar effect is observed when EC is completely replaced with EMC, the dissolution becomes negligible.

CV, UV/Vis spectroscopy, and ex situ SEM collectively confirm the insolubility of oxidized PVMPO in the various electrolytes. CV analysis demonstrates reversible redox behavior, with noticeable differences in current values, hinting at dissolution or slight variations in the charge compensation mechanism. The UV/Vis spectroscopy shows no detectable traces of the oxidized polymer even after 50 cycles, effectively ruling out solubility as the cause of the initial current fluctuations observed in CV. Additionally, SEM micrographs confirm suppressed dissolution and effective reversibility to the neutral state. These findings highlight the improved performance of the PVMPO electrode in the investigated electrolytes, which is further evaluated for long‐term electrochemical stability.

### 2.2 Electrochemical Performance

Galvanostatic charge–discharge (GCD) cycling, also referred to as constant current cycling (CCC), was conducted to evaluate the long‐term cycling stability of the PVMPO composite electrode at 1C rate using three different electrolytes: reference, EMC, and EMC + 1 wt.% FEC.

**Figure 4:**
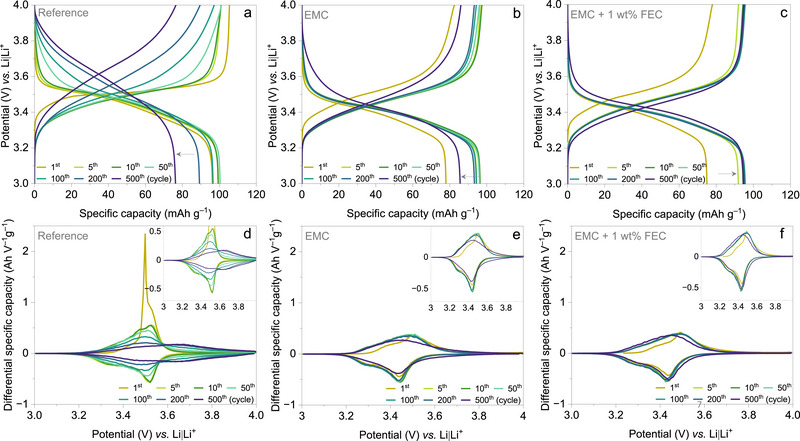
Electrochemical performance of PVMPO‐composite electrodes with three different electrolytes. a–c) Potential vs. capacity profiles for selected cycles using a) Reference, b) EMC, and c) EMC+ 1 wt.% FEC electrolytes. The grey arrow indicates the trend in capacity over the cycles, showing a decline for reference and EMC, while an increase is observed for EMC + 1 wt.% FEC during extended cycling. b,c) Differential specific capacity vs. potential profiles for selected cycles, with an inset showing a magnified view.

**Figure** **4** presents the potential vs. capacity curves with selected cycles at 1C rate. The cells utilizing the reference electrolyte delivered a first cycle discharge specific capacity of 96 mAh g^‒1^, which gradually increased to 100 mAh g^‒1^ by the end of 10 cycles. However, a steady decline in discharge specific capacity followed, reaching 76 mAh g^‒1^ at the end of 500 cycles, which is equivalent to 64% of the theoretical specific capacity. When referenced to the first cycle, the capacity retention is found to be 79%. After 100 cycles, the potential vs. capacity profile exhibits a slopy shape with the discharge potential increasing to 3.61 V vs. Li|Li^+^ (at the end of 500 cycles). This shift, along with the slopy nature, suggests a low redox activity in the polymer, contributing to the observed capacity fade over extended cycling (Figure 4a). Figure 4b presents the potential vs. capacity profiles for the cell utilizing the EMC electrolyte. The cell delivered an initial capacity of 78 mAh g^‒1^, which gradually increased to 96 mAh g^‒1^ by the 10^th^ cycle and remained stable up to the 50^th^ cycle. With continued cycling, the discharge specific capacity slightly declined to 93 mAh g^‒1^ at 200 cycles and further to 86 mAh g^‒1^ by the 500^th^ cycle. Relative to the first cycle, the capacity retention is 110%, due to the initially lower capacity compared to that at the 500^th^ cycle. When referenced to the more stable capacity observed at the 50^th^ cycle, the retention at 500 cycles is ≈89%. With the addition of 1 wt.% FEC to the EC‐free electrolyte (EMC + 1 wt.% FEC), the electrochemical performance enhances as shown in Figure 4c. The cells using the EMC + 1 wt.% FEC electrolyte delivered an initial discharge specific capacity of 75 mAh g^‒1^, which gradually increased to 92 mAh g^‒1^ by the 5^th^ cycle, 94 mAh g^‒1^ by the 10^th^ cycle and 95 mAh g^‒1^ by the 50^th^ cycle. This observed discharge specific capacity remained stable at 95 mAh g^‒1^ throughout the 500 cycles, indicating 100% capacity retention relative to the 50^th^ cycle. It is noteworthy that the use of EMC and EMC+ 1 wt.% FEC electrolytes does not result in a sloped potential vs. capacity profile during cycling. The potential vs. capacity curves maintain a nearly consistent shape throughout extended cycling, which is also evident from the corresponding differential specific capacity vs. potential curves derived from long‐term cycling measurements, as it provides information on the cell behavior and performance (Figure 4d–f). For the reference electrolyte, the high intensity peak indicates the electrolyte oxidation and anion insertion into the polymer structure. With extended cycling, the peak intensity gradually diminishes, broadens, and shifts to higher potentials after 200 cycles, indicating slower ion kinetics and lower redox activity, which is consistent with a previous study (Figure 4d and inset). In contrast, the EC‐free systems exhibit broader and less intense initial peaks compared to the reference system, indicating slower ion‐transport kinetics during the early cycles. However, with prolonged cycling, the peaks become more symmetric, reflecting stable redox reactions and improved cycling performance (Figure 4e,f, and corresponding insets).

Based on these findings, it is evident that the initial specific discharge capacities achieved with the EMC and EMC + 1 wt.% FEC electrolytes are 78 and 75 mAh g^‒1^, respectively, both notably lower than 96 mAh g^‒1^ observed with the reference electrolyte. However, this discrepancy diminishes over subsequent cycling. After 10 cycles, the specific discharge capacities for EMC and EMC + 1 wt.% FEC reach 96 and 94 mAh g^‒1^, respectively, which is close to 99 mAh g^‒1^ (reference). The difference observed during the initial cycles may be attributed to the elimination of EC from the electrolyte, as both the EMC and EMC + 1 wt.% FEC electrolyte systems exhibit similar behavior. However, the potential decreases to ≈3.4 V for both electrolytes. In contrast, the reference electrolyte exhibits a potential of 3.52 V in the early cycles.

To investigate whether these differences originate from the Li metal reference electrode, and to evaluate the impact of the electrolyte on the Li metal reference electrode as well as its possible influence on the cycling performance, the Li|Li^+^ reference electrode was calibrated in all three electrolytes using the ferrocene|ferrocenium ion (Fc|Fc^+^) redox couple as an internal reference.^[^ ^18^, ^19^ ^]^ The Fc|Fc^+^ redox potential is assumed to be stable in most solvents and electrolytes,^[^ ^20^ ^]^ any shift observed in the Fc|Fc^+^ potential would reflect a change in the potential of the Li reference electrode. However, no notable shift was observed for the electrolytes used in this study, as shown in Figure S2 (Supporting Information), indicating that the reference electrode remained stable and did not contribute to the observed potential shift in cells using different electrolytes.

Therefore, the reason for the shift in charge and discharge potential when using EC‐free electrolytes remains unclear. This reduction in discharge potential could be due to the nature of cathode electrolyte interphase (CEI) and some irreversible side reactions occurring on the surface of the PVMPO electrode. The subsequent increase in capacity to a maximum value suggests the stabilization of the CEI and improved accessibility of the polymer for PF_6_^‒^ during cycling, thereby improving ion transport.

**Figure 5:**
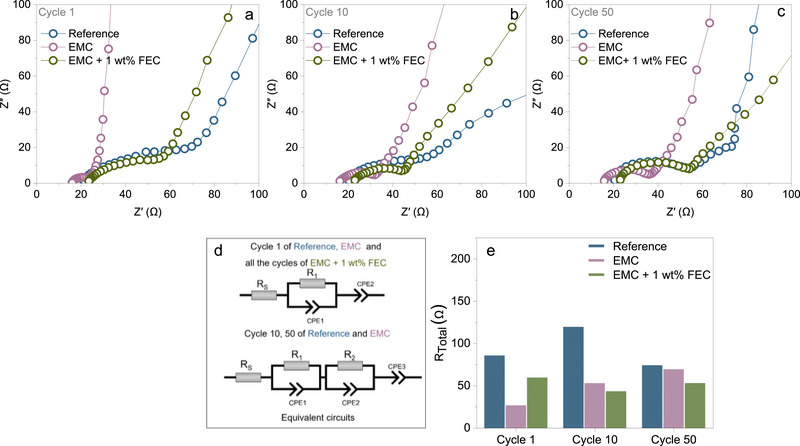
PEIS of PVMPO composite electrodes using three different electrolytes after the a) 1^st^ cycle, b) 10^th^ cycle, and c) 50^th^ cycle. The equivalent circuit model used for fitting is presented in (d). In graphs (a–c), the legends represent the experimental data points, while the lines represent the fitted results. Bar graph e) shows the total resistance of the cell at the end of the 1^st^, 10^th^, and 50^th^ cycle.

To further investigate the effect of different electrolytes on cell impedance in the initial cycles, in situ PEIS measurements were performed under the cell's operating conditions between cycles, while cycling at 1C rate. EIS, being a non‐destructive technique, enables monitoring of impedance changes without disturbing the system. **Figure** **5**a–c displays the Nyquist plot of the PVMPO electrodes after the 1^st^, 10^th^, and 50^th^ cycle, using the three different electrolytes. Figure 5d illustrates the equivalent circuit models used for fitting the impedance data, while Figure 5e shows a bar graph comparing the total resistance of the PVMPO electrodes with different electrolytes after the 1^st^, 10^th^ and 50^th^ cycle. **Table** **1** summarizes the resistance values obtained from the fitting.

**Table 1:** Summary of R_S_, R_1,_ and R_2_ values obtained from the fitting after the 1^st^, 10^th^, and 50^th^ cycle *(R_S_–solution resistance;* R_1_ and R_2_ could possibly be *interfacial/interphasial* and *charge transfer resistance*).

Electrolyte	Cycle 1 [Ω]			Cycle 10 [Ω]			Cycle 50 [Ω]		
	R_S_	R_1_	R_2_	R_S_	R_1_	R_2_	R_S_	R_1_	R_2_
Reference	19.4	66.3	–	18.4	42.6	58.5	19.1	35.8	19.2
EMC	15.4	11.3	–	15.6	17.7	19.7	14.9	20.9	33.7
EMC + 1 wt.% FEC	22.7	37	–	22.1	21.3	–	21.6	31.5	–

The R_S_ values exhibit negligible change upon extended cycling for all electrolytes investigated, indicating a stable ohmic resistance throughout the cycling process.

The impedance behavior of the PVMPO electrodes varied depending on the electrolyte formulation. The reference electrolyte exhibited higher interfacial and charge transfer resistance after the first cycle (66.3 Ω) compared to EMC (11.3 Ω) and EMC + 1 wt.% FEC (37 Ω). This elevated resistance in the reference system could be attributed to the higher viscosity of the electrolyte, which hinders charge transfer. Correspondingly, the Nyquist plots for the reference and EMC + 1 wt.% FEC systems displayed larger semicircles than those of EMC, indicating higher charge transfer resistance.

In the case of the reference electrolyte, interfacial and charge transfer resistance increased to 42.6 and 58.5 Ω after 10 cycles, which may reflect continued interfacial growth. By the 50^th^ cycle, the resistance decreased to 35.8 and 19.2 Ω, suggesting that the interface becomes more stable over extended cycling and facilitates improved charge transport. In contrast, the EMC electrolyte showed a gradual increase in interfacial and charge transfer resistance from 11.3 Ω initially to 37.4 Ω after 10 cycles (with 17.7 Ω interfacial and 19.7 Ω charge transfer resistance), and further to 54.6 Ω at 50 cycles (20.9 Ω interfacial and 33.7 Ω charge transfer resistance). Despite this increase, the resistance remained lower than that of the reference electrolyte throughout, indicating that the lower viscosity and thinner CEI layer in EMC promote better ion transport and reduced interfacial impedance.

The EMC + 1 wt.% FEC electrolyte demonstrated a different trend. Starting at 37 Ω (interfacial and charge transfer resistance), the resistance decreased to 21.3 Ω after 10 cycles, likely due to the formation of a more uniform and less resistive interface. However, by 50 cycles, the resistance rose to 31.5 Ω, possibly due to the thickening of the interface. The presence of FEC appears to enhance the stability of the interface layer, helping to suppress parasitic reactions and limit further electrolyte decomposition, which can contribute to an improved cycling performance. Additionally, for all three electrolyte systems, polymer structural rearrangements to accommodate anions also contribute to the observed charge transfer resistance. Nevertheless, EMC + 1 wt.% FEC generally maintained lower or comparable total resistance than the reference and EMC electrolytes over the extended cycles, suggesting improved interfacial stability (Figure 5e).

**Figure 6:**
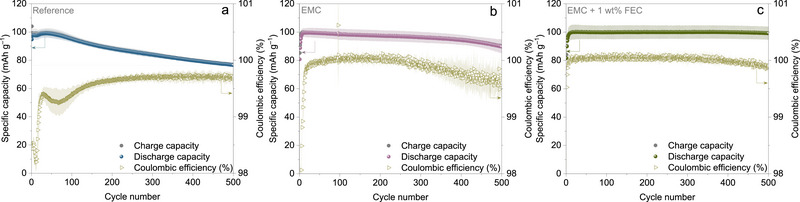
Long‐term cycling stability investigations of PVMPO composite electrodes cycled at 1C rate along with their corresponding CEs using a) Reference, b) EMC, and c) EMC + 1 wt.% FEC electrolytes.

**Figure** **6** shows the long‐term cycling stability and corresponding coulombic efficiencies (CEs) over 500 cycles at 1C rate. The CE across all three systems begins low, due to structural rearrangements in the polymer host to accommodate incoming guest ions (an activation phase). For the reference electrolyte (Figure 6a), CE gradually improves but remains inconsistent, indicating parasitic reactions during extended cycling. For EMC electrolyte (Figure 6b), CE stabilizes up to 200 cycles but later declines, indicating ongoing side reactions. By contrast, the EMC + 1 wt.% FEC electrolyte (Figure 6c) maintains high and stable CE, suggesting that the addition of FEC effectively suppresses side reactions, stabilizes the interface, and minimizes capacity degradation.

Additionally, the EC‐free electrolytes initially deliver a lower specific discharge capacity compared to the EC‐containing reference system, though the capacity gradually increases during the first cycles. This behavior indicates that the initial active site accessibility is limited but progressively improves upon cycling. This trend can be explained by electrolyte transport properties as well as interfacial chemistry. Removing EC lowers the viscosity, which should facilitate ion transport and improve ionic conductivity. However, in the absence of EC, the LiPF_6_ salt tends to form more contact ion pairs (CIPs) and aggregates rather than solvent‐separated ion pairs (SSIPs), resulting in lowered bulk conductivity.^[^ ^21^ ^]^ The addition of FEC partially mitigates this by solvating Li⁺, reducing ion pairing, and stabilizing the interface, which helps to recover conductivity losses.^[^ ^21^, ^22^ ^]^ Yet, this explanation stands in contrast to the observed capacity increase after a few cycles, which hints at a different interface formed with different electrolytes.

**Figure 7:**
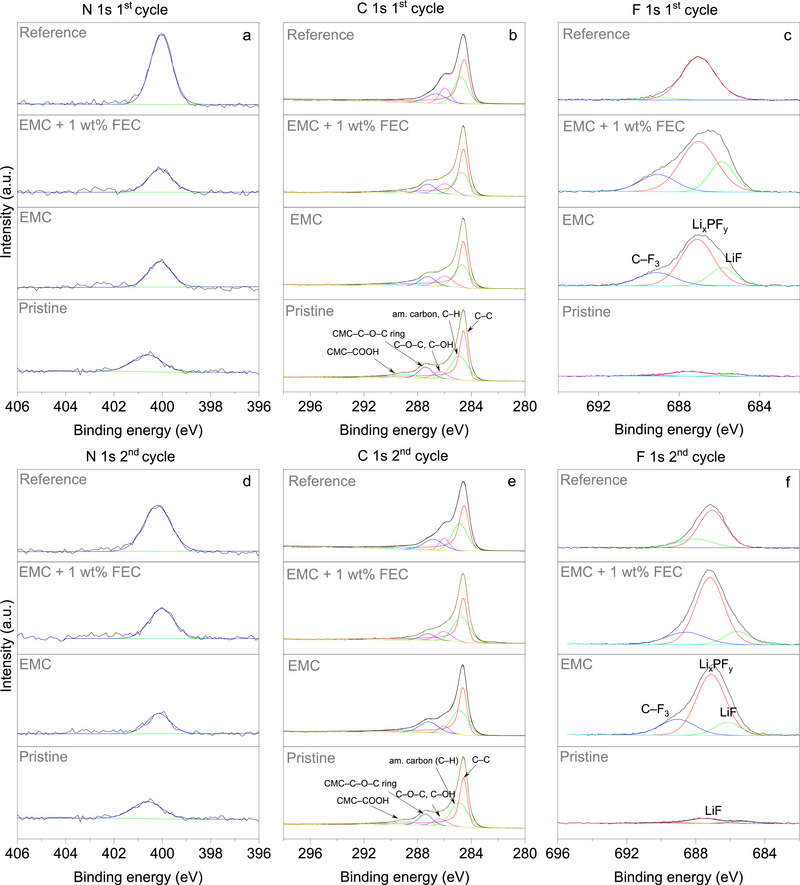
Normalized XPS spectra of pristine and cycled PVMPO electrodes using three different electrolytes after the 1^st^ and 2^nd^ cycle a, d) N 1s, b, e) C 1s, and c, f) F 1s regions.

To validate the assumption related to interfacial chemistry, XPS measurements were performed on the PVMPO electrodes after the 1^st^ and 2^nd^ cycle with various electrolytes, and the data are presented in **Figure** **7**. The average concentrations of the components are provided in Table S1 (Supporting Information) (pristine PVMPO electrode), Table S2 (Supporting Information) (1^st^ cycle) and Table S3 (Supporting Information) (2^nd^ cycle). A pristine PVMPO electrode was used as a reference for comparison.

Figure 7a,d presents high‐resolution N 1s spectra for pristine and cycled PVMPO electrodes in different electrolyte systems. All samples exhibit a characteristic peak near 400 eV, which can be attributed to the PVMPO. After cycling, a slight shift in peak position and a notable increase in intensity for the reference electrolyte (Tables S2 and S3, Supporting Information) are observed compared to the pristine electrode (Table S1, Supporting Information). The formation of an interfacial layer during the initial two cycles would reduce the N 1s signal intensity, an increase, however, indicates the PVMPO with migration to the surface. In the EC‐free systems (EMC and EMC + 1 wt.% FEC), this increase is notably lower. Therefore, this points to a strong interaction between PVMPO and EC, potentially increasing the exposure of nitrogen sites and contributing to higher electrochemical activity, consistent with higher initial discharge capacity. In contrast, in the EC‐free systems, the nitrogen signal is notably suppressed (Tables S2 and S3, Supporting Information), indicating reduced exposure of nitrogen sites. This is consistent with the observed lower initial capacity.

The C 1s spectra (≈284–290 eV) predominantly exhibit peaks corresponding to the binder, amorphous carbon, and C─O─C bonding in both pristine and cycled electrodes (Figure 7b,e). Notably, there are no distinct signals attributable to CEI‐related organic carbonate decomposition products, such as Li_2_CO_3_ or ROCO_2_Li,^[^ ^23^ ^]^ indicating minimal electrolyte breakdown and a negligible interfacial layer. However, for the reference electrolyte, the C─OH signal intensity is increased, which indeed indicates additional electrolyte decomposition reactions, which is not the case for the EC‐free electrolytes (Tables S2 and S3, Supporting Information).

The F 1s spectra presented in Figure 7c,f reveal peaks corresponding to LiF and Li_x_PF_y_ species, confirming a LiF‐rich CEI layer.^[^ ^23^ ^]^ By contrast, in the reference electrolyte, only the Li_x_PF_y_ peak is prominent, with no clear LiF signature. Interestingly, the intensity of those peaks is comparable for pure EMC and EMC + 1 wt.% FEC, indicating no substantial effect of the presence of FEC. The P 2p spectra presented in Figure S3 (Supporting Information) exhibit peaks ≈138 and 136 eV, corresponding to LiPF_6_ and LiPO_x_F_y_, which are the typical components of the SEI and CEI. Together, all these features suggest variations in CEI formation between the EC‐based and EC‐free electrolyte systems.

Taken together, the XPS results provide direct mechanistic validation of the cycling observations. In EC‐free electrolytes, a LiF‐rich CEI layer formed during the initial cycles limits the exposure of active sites, thereby lowering the initial capacity. This is supported by the suppressed N 1s intensity, compared to the EC‐based electrolytes. However, with continued cycling, polymer structural rearrangements and the stabilization of the CEI enhance active site exposure, thereby accounting for the observed capacity recovery.

The results also indicate that the addition of FEC did not lead to a different CEI; hence the observed improved capacity retention is rather related to improved SEI formation, reducing cross‐talk and capacity fade. It is well established that FEC forms a more stable and elastic LiF‐rich SEI on lithium metal negative electrodes. This inhibits lithium dendrite growth and delays parasitic reactions, thereby suppressing electrolyte‐lithium cross‐talk that could otherwise promote electrode degradation.^[^ ^24^, ^25^, ^26^ ^]^ In contrast, while EC‐based electrolytes (reference) can form an unstable SEI and dendrites on the lithium side, facilitating electrode decomposition. However, EMC alone is not capable of forming a stable SEI on the lithium counter electrode. This limitation can be reflected in cell performance, as demonstrated by Li et al., where graphite‖Li half‐cells using only EMC exhibit severe capacity fading.^[^ ^27^ ^]^ With the addition of FEC, EMC‐based electrolyte without EC can form a more stable SEI. Nonetheless, in the present study, long‐term cycling of cells with an EMC electrolyte, even without FEC shows remarkable stability over 500 cycles, with only minor capacity decay. The addition of FEC further enhances performance by providing greater stability and leading to higher accumulated capacity, with minimal signs of parasitic reactions. Thus, these effects are more closely associated with SEI formation than with CEI. These observations indicate that, under the studied conditions, the lithium counter electrode and its SEI have minimal impact on overall performance in EMC‐only systems. However, when FEC is added, the SEI becomes a substantial factor contributing to improved performance.

**Figure 8:**
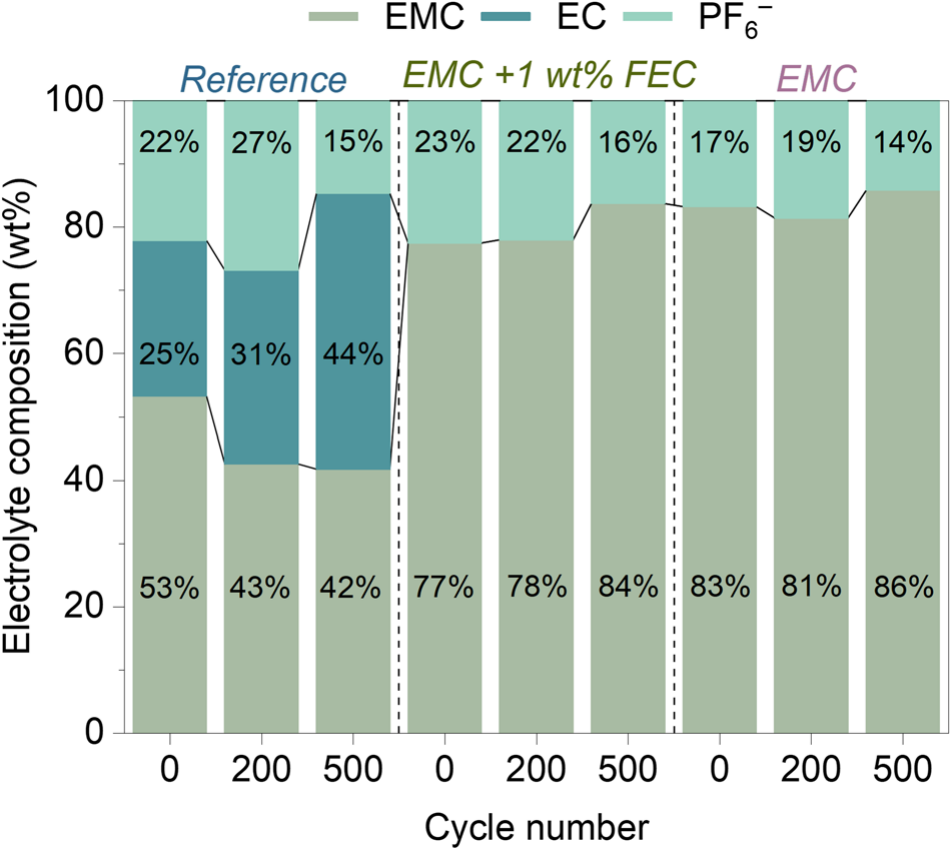
Combined IC‐CD and GC‐FID analyses of electrolyte composition evolution in reference, EMC, and EMC + 1 wt.% FEC systems over 500 cycles. Relative concentrations of EMC, EC, and PF_6_^−^ reveal progressive EMC depletion in reference, nonmonotonic PF_6_^−^ redistribution dynamics, and suppressed degradation in FEC‐containing electrolytes due to interface stabilization.

Electrolyte stability plays a critical role in determining the long‐term performance and lifetime of dual‐ion batteries. While the cycling behavior and XPS analysis display differences across the three electrolytes, changes in the electrolyte composition of various electrolyte systems were quantitatively analyzed throughout cycling. **Figure** **8** displays the analysis results of the electrolytes extracted from the separator of both pristine and cycled cells.

For the cells using a reference electrolyte, a decrease in the relative amount of EMC over time is observed. In parallel, the PF_6_^−^ concentration shows a nonmonotonic trend after 200 cycles, it initially increases from 22 to 27 wt.%, followed by a decrease to 15 wt.% after 500 cycles. This behavior can be explained through multiple ways. 1) oxidative decomposition at the high‐voltage organic positive electrode interface, and 2) chemical reactions with oxidized PVMPO surface sites generating reactive intermediates that catalyze solvent decomposition, leading to relative PF_6_^–^ enrichment in the remaining electrolyte and 3) possible intercalation of linear carbonate molecules with the polymer matrix, leading to apparent PF_6_^−^ enrichment in the remaining electrolyte.^[^ ^28^, ^29^ ^]^ The subsequent decrease in PF_6_^–^ concentration to 15 wt.% suggests irreversible salt consumption. PF_5_ species generated from LiPF_6_ decomposition may react with polymer, leading to irreversible salt loss. Additionally, the ongoing polymer‐electrolyte interface evolution promotes electrolyte‐polymer interactions, consuming both salt and solvent components.^[^ ^28^ ^]^

Cells using EMC as the electrolyte generally show lower relative PF_6_^−^ concentrations compared to other systems. After 500 cycles, a slight decrease in relative PF_6_^−^ concentration is observed from 17 to 14 wt.%. However, the EMC content remains highly stable, ranging between 83 and 86 wt.%. In pure EMC electrolyte, the linear carbonate solvent molecules can co‐intercalate along with an anion, into the PVMPO polymer structure, as described earlier. This co‐intercalation process can dynamically alter the local electrolyte composition within the polymer matrix. The initial PF_6_^–^ increase (0–200 cycles) could result from preferential EMC uptake by the polymer, concentrating the remaining salt in the bulk electrolyte phase. Such solvent intercalation may cause structural expansion and electrode degradation during cycling.^[^ ^30^, ^31^ ^]^

Cells with EMC + 1 wt.% FEC electrolyte exhibit a remarkable stability in electrolyte composition during the first 200 cycles. The relative concentrations of EMC (77–78 wt.%) and PF_6_^−^ (22–23 wt.%) remain nearly unchanged. After 500 cycles, the PF_6_^−^ concentration drops to 16 wt.%, while the EMC content increases to 84 wt.%. The gradual PF_6_^−^ decrease in the FEC system represents controlled electrolyte aging without the complex solvent intercalation‐deintercalation cycles. As supported by XPS, FEC addition does not alter the CEI composition, but forms a more stable, LiF‐rich SEI at the lithium electrode that reduces cross‐talk between electrodes. With suppressed EMC penetration into the polymer matrix, the electrolyte composition remains more stable, and salt consumption occurs primarily via SEI formation rather than through polymer‐solvent interactions.

Identification of the compounds was confirmed using GC‐MS. Due to the low FEC content and the undergoing film‐forming reaction by FEC, it could neither be detected via GC‐MS nor GC‐FID.^[^ ^32^ ^]^ Table S4 (Supporting Information) summarizes the changes in electrolyte composition over 500 cycles, while Figure S4 (Supporting Information) displays the GC‐EI‐MS analysis of both the pristine and *post*‐postcycled electrolytes.

These electrochemical and compositional analyses demonstrate that the addition of 1 wt.% FEC to EC‐free electrolyte systems notably enhances electrochemical stability, minimizes impedance growth during initial cycles, and maintains a stable electrolyte composition over extended cycling. These improvements surpass the performance of both the reference and EMC‐only electrolyte systems. This enhanced performance can be primarily attributed to the formation of a robust SEI, which suppresses parasitic side reactions, improves reversibility, and ultimately extends cycle life while providing more accessible capacity and better capacity retention.

### 2.3 Degradation Mechanism Insights Using Ex situ SEM, EDS, and LSM Micrographs

**Figure 9:**
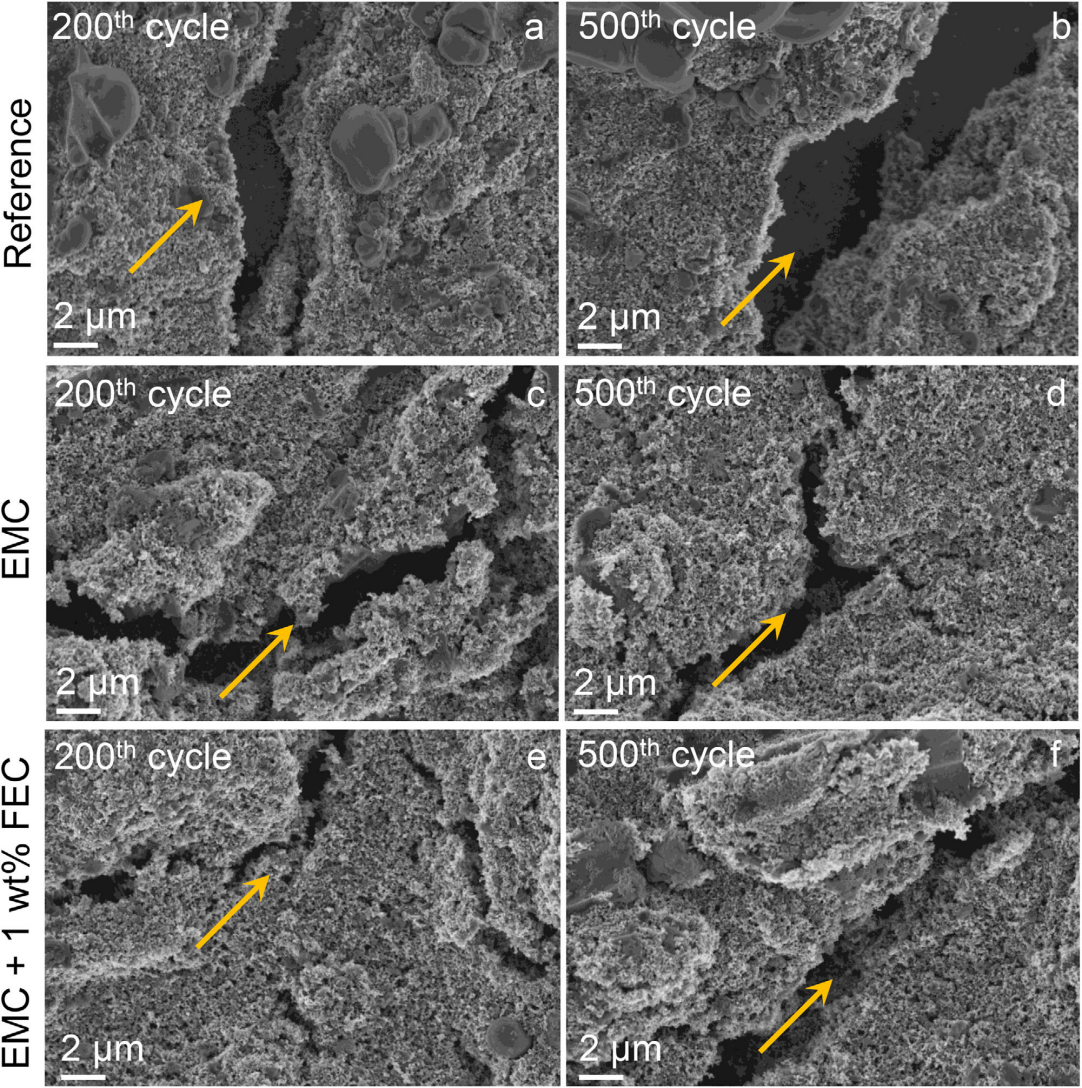
Ex situ SEM images of PVMPO electrodes after the 200^th^ and 500^th^ cycle, cycled using different electrolytes. (a, b) Reference, (c, d) EMC, and (e, f) EMC + 1 wt.% FEC.

Further, *post*‐cycling analyses were conducted to evaluate the physical changes in the cycled electrodes, lithium metal, and separators. **Figure** **9** presents ex situ SEM images of the electrodes after 200 and 500 cycles with different electrolytes.

It is evident that prolonged cycling causes crack formation (highlighted by yellow indicators), which becomes more severe with increased cycling, particularly in the electrodes without FEC (Figure 9a–d). This leads to a loss of contact between the active material, reducing electronic conductivity and contributing to capacity loss over extended cycling. Notably, in the electrodes cycled with EMC + 1 wt.% FEC (Figure 9e,f), the extent of cracking is markedly reduced after 500 cycles, suggesting that FEC contributes to the stabilization of the electrode microstructure that minimizes decomposition or mechanical degradation. Thus, the electrode maintains better structural integrity, improved specific capacity, and enhanced cycle life.

**Figure 10:**
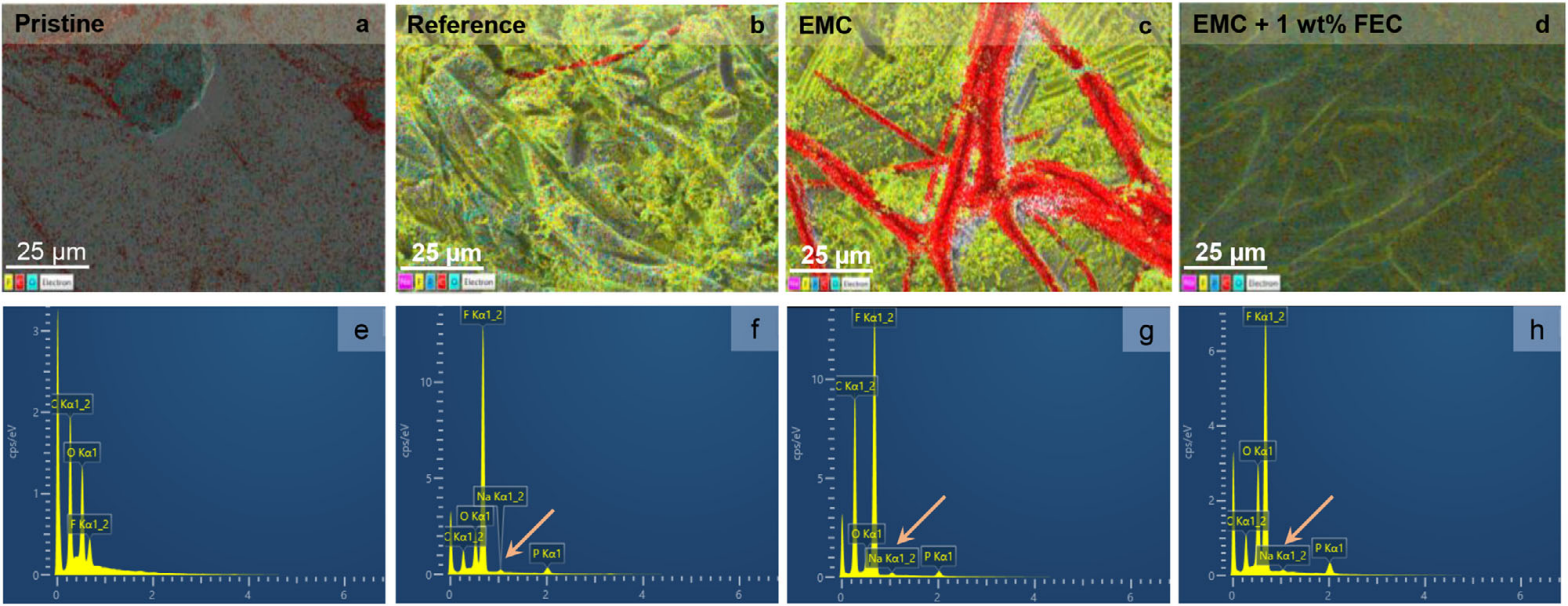
EDS mapping and spectra of pristine and cycled lithium after 500 cycles in three electrolytes. a, e) Pristine lithium, b, f) Reference, c, g) EMC, and d, h) EMC + 1 wt.% FEC. Pale orange arrows highlight sodium signals. Elemental mapping colors: Carbon (C)—red, Sodium (Na)—magenta, Phosphorous (P)—blue, and Oxygen (O)—cyan.

Furthermore, black‐colored deposits were observed on the lithium surface after cycling. These deposits were analyzed using SEM and EDS measurements. Figure S5 (Supporting Information) displays the SEM images of lithium cycled with different electrolytes after 500 cycles. **Figure** **10** displays the EDS mapping of cycled lithium after 500 cycles, alongside pristine lithium and the corresponding elemental spectra.

EDS mapping of pristine lithium shows distinct peaks for carbon (C), fluorine (F), and oxygen (O). In contrast, cycled lithium exhibits additional signals attributed to the decomposition products of the electrolyte, as well as minor traces from the PVMPO electrode. Notably, sodium, originating from the binder, is detected and marked by orange indicators, along with phosphorus, fluorine, carbon, and oxygen, which are characteristic markers of electrolyte degradation (Figure 10a–d). The EDS spectra reveal that the intensity of sodium on the lithium surface in the EMC + 1 wt.% FEC system (Figure 10d) is relatively lower or nearly negligible, when compared to the sodium signals observed with the reference and EMC electrolytes (Figure 10b,c). This reduction in sodium presence strongly indicates that electrode decomposition is comparatively suppressed when using the EMC + 1 wt.% FEC electrolyte.

However, no nitrogen signal, a key for identifying the polymer, was observed on the lithium surface. The polymer structure is provided in Figure S6 (Supporting Information). This absence suggests that the polymer may have remained confined to the electrode surface, dissolved into the electrolyte, or possibly be present on the separator.

Further UV/Vis‐spectroscopic measurements were carried out after 500 cycles for all three electrolytes, and the data presented in Figure S7 (Supporting Information) suggest that no dissolution occurs upon prolonged cycling. Similar to the observations made on the lithium counter electrode, black deposits were also found on the separators after 500 cycles in all three electrolytes. LSM micrographs of both the pristine and cycled separators are provided in Figure S8 (Supporting Information), showing the presence of deposits on the separators, which could be attributed to polymer residues and decomposition products of the electrolyte.

In summary, *post* *‐*cycling analyses strongly support earlier findings that the addition of FEC effectively suppresses parasitic side reactions and electrode decomposition, leading to enhanced cycle life and increased accessible capacity.

## 3 Conclusion

This study investigates the role of EC‐free electrolyte systems on the electrochemical performance of organic redox polymers, particularly PVMPO. The solubility of the oxidized state presents a key challenge that compromises long‐term stability and thus cycling stability. Prior studies have shown that reducing the EC content and employing higher concentrations of bulkier solvents can mitigate this issue. Building on these findings, this study explores the use of an EC‐free electrolyte, 1.0 m LiPF_6_ in EMC with and without 1 wt.% FEC as an additive.

To our knowledge, this is the first systematic study evaluating the electrochemical behavior of redox polymers in EC‐free electrolyte systems and assessing the role of FEC in such formulations for organic electrodes. The findings suggest that EC‐free electrolyte systems markedly inhibit the dissolution of PVMPO, as confirmed by CV, UV/Vis spectroscopy, and ex situ SEM analyses conducted during the initial cycles. Compared to the reference electrolyte (1.0 m LiPF_6_ in 3:7 EC:EMC) which show a gradual capacity fading and retained 79% of PVMPO's capacity after 500 cycles (76 mAh g^−1^), EC‐free systems using EMC alone or EMC with 1 wt.% FEC yield improved discharge specific capacities of 86 and 95 mAh g^−1^, corresponding to 72% and 79% of the theoretical capacity, and 89% and 100% capacity retention (normalized to the 50^th^ cycle), respectively.

The enhanced performance of the EMC + 1 wt.% FEC system is attributed to the formation of a more stable and protective interfacial layer, which helps suppress parasitic side reactions and supports prolonged cycle life and capacity retention. *Post‐*cycling SEM imaging further demonstrates that electrodes cycled with FEC exhibit fewer cracks after 500 cycles, preserving electrode integrity. Conversely, electrodes without FEC develop severe microstructural degradation, compromising conductivity and contributing to capacity loss. EDS analysis of lithium counter electrodes reveals minimal trace migration of the active polymer in FEC‐containing systems, indicating reduced dissolution and cross‐contamination. Supporting this, UV/Vis spectroscopy detected no signal for the oxidized polymer in the electrolyte, suggesting that the polymer predominantly remains on the separator rather than migrating to the lithium electrode. LSM images of separators after 500 cycles showed fewer deposits in the EMC + FEC system compared to the reference or EMC‐only electrolytes.

In summary, this work demonstrates that EC‐free electrolytes, especially when combined with FEC, offer a promising route to improve the stability and performance of organic redox polymer‐based batteries. The results highlight the importance of electrolyte design in mitigating dissolution, reducing parasitic reactions, and preserving electrode integrity, thus paving the way for the development of more robust and long‐life organic energy storage systems.

## 4 Experimental Section

### Synthesis of PVMPO

The synthesis and characterization of PVMPO were previously studied and reported elsewhere.^[^ ^11^ ^]^

### Fabrication of PVMPO Electrodes

Electrodes were prepared by mixing the as‐synthesised PVMPO powder, the commercially available conductive additive carbon black (Super C65, Imerys Graphite & Carbon), and sodium carboxymethyl cellulose (Na‐CMC, CRT 2000 PPA12 Dowwolff Cellulosics, Germany) in a Thinky mixer (Arm‐310) for 1 h at 1700 rpm in the weight ratio of 50:45:5, respectively. Initially, the solid components were hand‐mixed using a mortar and pestle for 30 min followed by the addition of the binder solution and the required amount of water in the same weight ratio (50:45:5). Once the required electrode paste consistency was achieved, the mixture was mixed in the Thinky mixer for 1 h at 1700 rpm. The electrode pastes obtained were cast on 5 wt.% KOH‐etched aluminum foil by doctor blade coating (50 µm spacing). The resulting electrodes were dried in a hot air oven for 1 h (primary drying) and a vacuum drying cabinet at 70 °C overnight (secondary drying). Electrode discs of 12 mm diameter were prepared, pressed in a hydraulic press at 3 tons pressure for 20 s, and further dried at 120 °C under reduced pressure of 0.005 mbar for 12 h in a Büchi B‐585 glass oven. The resulting electrodes had dry film thicknesses of 10–13 µm (excluding the thickness of the current collector) and active mass loadings between 0.19–0.35 mg cm^‒2^.

### Preparation of Electrolytes

Lithium hexafluorophosphate (LiPF_6_, Elyte) was employed as the conducting salt, while ethylene carbonate (EC), ethyl methyl carbonate (EMC) (both from Elyte), and fluoroethylene carbonate (FEC, BASF) were used as solvents. All chemicals were used as received without further purification. Three electrolyte formulations were prepared for this study: 1.0 m LiPF_6_ in a 3:7 (w/w) mixture of EC and EMC (referred to as the “Reference” electrolyte), 1.0 m LiPF_6_ in EMC (referred to as the “EMC” electrolyte), and 1.0 m LiPF_6_ in EMC with 1 wt.% FEC (referred to as the “EMC + 1 wt.% FEC” electrolyte). For each formulation, the solvent components were mixed overnight using magnetic stirring. LiPF_6_ and, where applicable, FEC were then added to the solvent mixture, followed by further overnight stirring to ensure complete dissolution and homogeneity of the final electrolyte. Electrolyte preparation was conducted in an MBraun glovebox under an argon atmosphere, with water and oxygen levels maintained below 0.01 ppm. Prior to use, the electrolytes were dried overnight over molecular sieves that had been activated at 300 °C for 24 h under reduced pressure. For the calibration of the Li metal RE, ferrocene (ThermoFischer Scientific, 99%) was dissolved in the electrolytes prior to cell assembly.

### Chemicals for Ion Chromatography (IC), Gas Chromatography‐Mass Spectrometry (GC‐MS) and Gas Chromatography‐Flame Ionization Detector (GC‐FID) Measurements

Hypergrade acetonitrile (ACN) was purchased from Merck KGaA. Ultrapure water (H2O) was filtered with a Millipak 20 filter having a pore size of 0.22 µm that was installed into a Millipore Milli‐Q system (Bendford, USA). Dichloromethane (DCM), fluorethylene carbonate (FEC), and potassium hexafluorophosphate were purchased from Sigma‐Aldrich.

### Cell Assembly and Electrochemical Measurements

The electrochemical performance of the PVMPO composite electrodes was investigated using a three‐electrode Swagelok T‐Cell setup. The six layers of Freudenberg 2190 nonwoven PP separator (FS‐2190) soaked with 130 µL electrolyte were used for the counter electrode and the PVMPO composite electrode (13 mm separator) and 60 µL (10 mm separator) for the reference electrode. The separators used were punched and pre‐dried under reduced pressure of 0.005 mbar at 120 °C for 24 h in Büchi B‐585 glass oven before cell assembly. Galvanostatic cycling investigations were performed in half cells using lithium metal as counter (12 mm disc) as well as a reference electrode (5 mm disc). The cells were assembled in a dry room with less than 20 ppm humidity and given a rest time of 12 h before cycling. They were cycled at a 1C rate assuming a theoretical capacity of 120 mAh g^‒1^ (considering a one‐electron redox process) operated in the voltage range of 3.0–4.0 V. Cyclic voltammetry (CV) was performed with Swagelok T‐cells using Biologic (VMP3 Potentiostat) Science Instruments at 20 °C in a Binder oven between the voltage window of 3.0 to 4.0 V. To calibrate the Li‐metal reference electrode, the working electrode was substituted with a platinum (Pt) electrode (12 mm diameter), while lithium was used as both the counter and reference electrode. Electrolytes containing dissolved 0.05 m ferrocene were employed, and the potential of ferrocene was determined from CV measurements performed at a scan rate of 0.1 mV s^‒1^, under the same temperature and voltage conditions using the Biologic VMP3 Potentiostat. In situ PEIS (Potentiostatic Electrochemical Impedance Spectroscopy) measurements were carried out under the cell's operating conditions at different cycles. The cell setup, similar to that used in other cycling experiments, was employed. The measurements were conducted across a frequency range of 1–20 mHz, with an amplitude of 10 mV using a Biologic (VMP3 Potentiostat) Science Instruments system at 20 °C inside a Binder oven.

### Scanning Electron Microscopy (SEM)

SEM micrographs of the PVMPO composite electrodes (pristine as well as ex situ) were measured on a Zeiss Crossbeam 550 electron microscope (Carl Zeiss Microscopy GmbH) at an accelerating voltage of 3 kV using an in‐lens detector at a working distance of 4 mm. For ex situ measurements, the cells were disassembled in a glove box and the samples were transferred using a vacuum‐sealed sample holder to avoid exposure to air. For the lithium metal SEM measurements, the working distance maintained was 5 mm. For Energy Dispersive X‐ray Spectroscopy (EDS), the accelerating voltage used was 6 kV and working distance maintained was 5 mm.

### X‐ray Photoelectron Spectroscopy (XPS)

X‐ray photoelectron spectroscopy (XPS) analysis was conducted using a Kratos AXIS Ultra DLD spectrometer (UK), equipped with a monochromatic Al Kα (1486 eV) X‐ray source, operating at an emission current of 10 mA and an accelerating voltage of 12 kV. After electrochemical cycling, the cells were disassembled inside an argon‐filled glove box, and the samples were transferred to the instrument in a sealed container to avoid air exposure. An integrated argon glove box was used for direct sample insertion. Prior to analysis, the samples were maintained in the instrument's ultra‐high vacuum chamber for over 12 h to ensure the removal of volatile components. The pressure in the analysis chamber was maintained at ≤5 × 10^‒8 ^mbar. A charge neutralizer was employed to compensate for surface charging during measurement. For each analysis, five individual scans were recorded to check for differential charging, and identical spectra were subsequently averaged to improve the signal‐to‐noise ratio. All core‐level spectra were acquired at a pass energy (PE) of 20 eV and calibrated against the C–C peak at 284.6 eV. Two independent electrodes from each sample were analyzed, with measurements taken at two different locations on each electrode. Given only minor variations between measurements, the combined data sets were used for representation.

### Laser Scanning Microscopy (LSM)

LSM micrographs were obtained using a VK‐X260 measuring unit in combination with a VK‐X250 control device. Surface imaging was conducted with a 10× magnification lens and a 408 nm laser source. Data analysis was carried out using MultiFile Analyzer software.

### UV/Vis Spectroscopy

UV/Vis‐spectroscopic measurements of the electrolytes were measured with a Shimadzu UV‐2450 spectrometer using a sealed quartz glass cuvette (115‐QS, Hellma Analytics) with a path length of 10 mm from 200 to 800 nm. The charged and discharged cells were disassembled in the glove box (O_2_ ˂0.1 ppm; H_2_O ˂0.1 ppm) and the separator was placed in a 1.5 mL Safe‐Lock Tube with a spacer (Eppendorf). The sealed lock tube was placed in the centrifuge (Galaxy SD Microcentrifuge, VWR International GmbH, Germany), and the electrolyte was extracted from the separator at 8500 rpm for 5 min. Then, 10 µL of the electrolyte extracted was diluted with 1000 µL (1 mL) of acetonitrile (ACN) in the quartz vial for measurements.

### Ion Chromatography (IC), (GC‐MS) and Gas Chromatography‐Flame Ionization Detector (GC‐FID) Measurements

Ion chromatography (IC) was performed on an 850 Professional IC (Metrohm, Herisau, Switzerland) with conductivity detection (CD). A Metrosep A Supp 7‐ (250 × 4.0 mm, 5 µm; Metrohm) with a Metrosep A Supp 4/5 guard column was used for isocratic anion separation at 60 °C and a flow rate of 0.7 mL min^‒1^ was applied. The developed method is based on Kraft et al.^[^ ^33^ ^]^ Further parameters and sample preparation were applied according to Henschel et al.^[^ ^34^ ^]^ The electrolyte was diluted using ACN 1 to 2000.

The Gas Chromatography‐Flame Ionization Detector (GC‐FID) experiments were executed on a Shimadzu Nexis GC‐2030 (Nakagyo‐ku, Japan) equipped with a Restek Rxi‐5 ms (30 m × 0.25 mm, 0.25 µm; Restek GmbH, Bad Homburg, Germany) diphenyl dimethyl polysiloxane (5%/95%) fused silica column. Further parameters and sample preparation were conducted according to Terborg et al.^[^ ^35^ ^]^ To achieve analyte concentrations within the linear measuring range of the GC‐FID and IC‐CD calibrations, the electrolytes had to be diluted. An overall dilution factor of 1:5000 was required, which was achieved in two gravimetric steps (1:100 and 1:50) using ACN. For electrolytes containing FEC, a dilution factor of 1:1000 (1:100 and 1:10) was used due to the lower initial concentrations of the additive. 1 µL was injected at 250 °C with a split ratio of 1:100. The system was run with a column flow of 1.16 mL min^‒1^ Helium, a purge flow of 3 mL, and the following column oven program: starting at 40 °C for 1 min, the temperature was increased by a rate of 3 °C min^‒1^ up to 60 °C, followed by a second ramp with a rate of 30 °C min^‒1^ up to 230 °C, held for 1 min. The FID detector was set to a temperature of 260 °C with a hydrogen flow of 32 mL min^−1^, an air flow of 200 mL min^‒1^, and a makeup flow of 24 mL min^‒1^.

Gas Chromatography‐Electron Ionization‐Mass Spectrometry (GC‐EI‐MS) experiments were executed on a Shimadzu GC‐MS QP2010 Ultra with an assembled AOC‐5000 Plus autosampler and a nonpolar Supelco SLB‐5 ms (30 m × 0.25 mm. 0.25 µm; Sigma Aldrich Chemie GmbH, Steinheim, Germany) column. Further parameters and sample preparation conditions were applied according to Grützke and Mönninghoff et al.^[^ ^36^, ^37^ ^]^ The electrolyte was diluted using DCM 1 to 10 000. The 1 µL was injected at 250 °C with a split ratio of 1:100. The system was run with the same GC parameters as for the FID measurements. The MS was operated in EI ionization mode with an ion source temperature of 200 °C, an interface temperature of 250 °C, a filament voltage of 70 V and an event time of 0.1 s in the range of m/z 10–400. The detector voltage was set relative to current tuning results (≈ 1 kV).

## Conflict of Interest

The authors declare no conflict of interest.

## Author Contributions

S.P.P. fabricated the electrodes, conducted the electrochemical investigations, data analysis, and drafted the original manuscript (except the IC‐CD, GC‐FID and GC‐MS analysis). S.A. synthesized the polymer PVMPO under the supervision of B.E. J.M.H. performed and analyzed the IC‐CD, GC‐FID and GC‐MS measurements under the supervision of S.N. U.R. carried out and fitted the XPS measurements. M.W. and P.B. supervised the work and corrected the manuscript. S.A., J.M.H., and B.E. also reviewed the manuscript. All authors discussed, revised, and approved the final version of the manuscript.

## Supporting information



Supporting Information

## Data Availability

Data are available from the corresponding author on request.
