# Supplementary material for: Improving Cycle Life and Capacity Retention in PVMPO‖Li Dual‐Ion Lithium‐Organic Batteries Using an EC‐Free and FEC Additive Containing Electrolyte
Source: Small Methods. 2026 Jan 14;10(3):e01766. doi: 10.1002/smtd.202501766 (PMC12893304; doi:10.1002/smtd.202501766)
Supplement: Supplementary file 1 — Supporting Information [file SMTD-10-e01766-s001.docx]

Improving Cycle Life And Capacity Retention In PVMPO‖Li Dual-Ion Lithium-Organic Batteries Using an EC-Free And FEC Additive Containing Electrolyte

Sathiya Priya Panjalingam,^1,2^ Somayeh Ahadi,^3^ Jakob Hesper,^1^ Uta Rodehorst,^1^ Sascha Nowak,^1^ Birgit Esser,^3^ Martin Winter ^1,4^ and Peter Bieker*^1,4^

Corresponding author: [peter.bieker@fz-juelich.de](mailto:peter.bieker@fz-juelich.de)

^1^ *MEET Battery Research Center, Institute of Physical Chemistry, University of Münster,*

*Corrensstr. 46, 48149 Münster, Germany*

^2^ *International Graduate School for Battery Chemistry Characterization Analysis Recycling*

*and Application (BACCARA), Corrensstr. 40, 48149 Münster, Germany*

^3^ *Institute of Organic Chemistry II and Advanced Materials, Ulm University, Albert-Einstein-Allee 11, 89081, Ulm, Germany*

*^4^ Helmholtz Institute Münster (HI MS), IMD-4, Forschungszentrum Jülich Gmbh, University of Münster, Corrensstr. 46, 48149 Münster, Germany*

**Figure S1.** *Ex situ* SEM micrographs of PVMPO composite electrodes after 10^th^ and 50^th^ cycles in (a, b) Reference (c, d) EMC and (e, f) EMC+ 1 wt% FEC electrolytes, respectively.


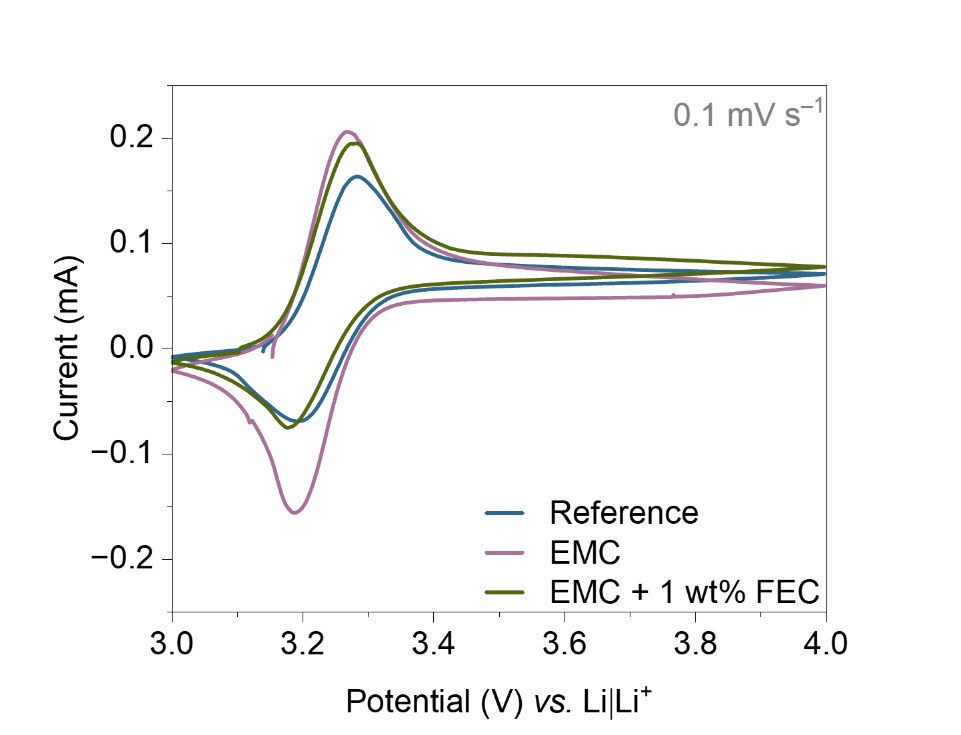


**Figure S2.** Cyclic voltammograms (CVs) of Pt‖Li Swagelok cells in a three-electrode configuration (Li serving as both, counter and reference electrode), recorded with 0.05 m ferrocene added to three different electrolytes.

**Figure S3:** Normalized XPS spectra of the P 2p regions of pristine and cycled PVMPO electrodes, using three different electrolytes, after the 1^st^ and 2^nd^ cycle.


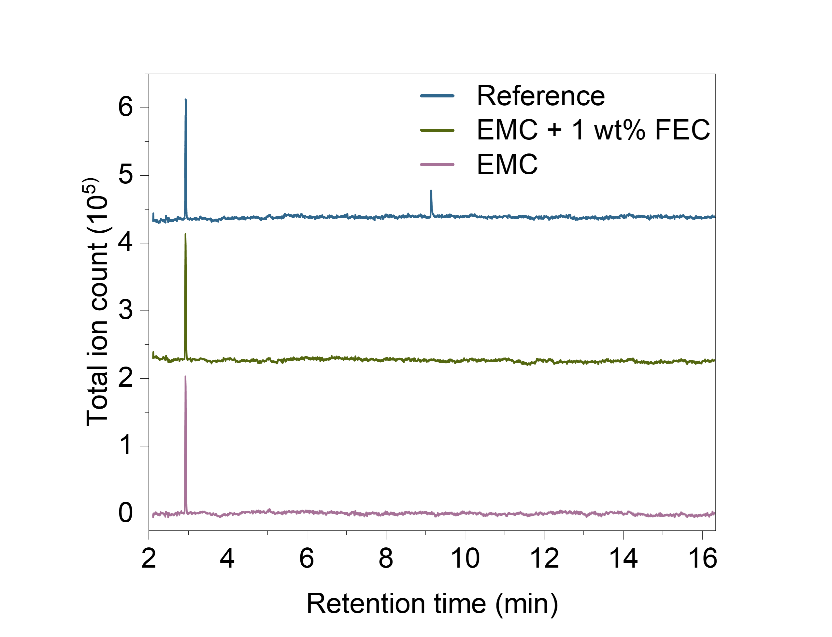


**Figure S4**. Blank adjusted GC-EI-MS chromatograms of the pristine electrolytes diluted one to 100 in DCM. EMC was identified at a retention time of 3.0 min and EC at a retention time of 9.1 min. FEC could not be detected.

**Figure S5.** SEM images showing (a) Pristine lithium and lithium after 500 charge-discharge cycles using three different electrolytes: (b) Reference, (c) EMC, and (d) EMC with 1 wt% FEC additive. Yellow arrows indicate the deposits on the lithium counter electrode surface.


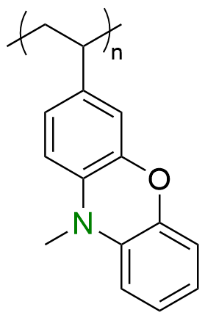


**Figure S6.** Chemical structure of the PVMPO polymer

**Supporting Figure S7** shows the UV/Vis spectra of all three electrolytes after 500 cycles. Across all three systems, no evidence of dissolution of the oxidized state of PVMPO was detected even after 500 cycles, as indicated by the absence of an absorption band around 540 nm—the characteristic signal of the oxidized state of PVMPO. This region is highlighted with a grey ellipse, confirming the absence of dissolution of the oxidized state **(Supporting Figure S7a).**

**Figure S7.** UV/Vis spectra of the three different electrolytes extracted after 500 cycles (a)full wavelength range from 200 to 800 nm, and (b) magnified view of the 200 to 300 nm region.

On the other hand, the intensity of the peaks around ≈230 nm showed a slight increase for all three electrolyte systems after 500 cycles compared to the UV/Vis measurements from the initial cycles **(Figure 2).** The peaks at ≈ 236 nm for the reference, ≈ 229 nm for EMC, and ≈ 231 nm for EMC + 1 wt% FEC electrolytes, may correspond to the neutral PVMPO polymer, suggesting the presence of the neutral polymer in the electrolyte **(Supporting Figure S7b).** However, it does not appear to migrate to the lithium counter side and instead remains may be present on the separator.

**Supporting Figure S8** presents LSM images of the pristine separator **(Figure S8a)** and those after 500 cycles using the three different electrolytes **(Figures S8 (b–d)).**

**Figure S8.** LSM micrographs of (a) Pristine and cycled separators after 500 cycles using three different electrolytes (b) Reference, (c) EMC and (d) EMC + 1 wt% FEC. The yellow boxes highlight the depositions found on the separator.

In contrast to the pristine separator, which exhibits a clean and well-defined micro-fibrous structure, all the cycled separators show surfaces covered with deposits. These deposits are most likely the result of polymer residues and decomposition products of the electrolyte formed during cycling. The extent and morphology of the deposits vary depending on the electrolyte formulation. Notably, the separator cycled in the EMC + 1 wt% FEC electrolyte **(Figure S8d)** shows comparatively fewer deposits and retains more of the original morphology of the pristine separator compared to the separators cycled in reference and EMC electrolyte
**(Figure S8b and S8c).** This further suggests that the FEC additive helps suppress electrolyte degradation and electrode decomposition.

**Table S1.** Relative atomic concentrations of pristine PVMPO electrodes.

| Components | Pristine PVMPO electrode | |
| --- | --- | --- |
|  | Avg. Conc. (%) | SD (%) |
| N 1s | 0.82 | 0.06 |

**Table S2.** Relative atomic concentrations of PVMPO electrodes after 1^st^ cycle with three different electrolytes.

| Components | Electrolyte (after 1^st^ cycle) | | | | | |
| --- | --- | --- | --- | --- | --- | --- |
|  | Reference | | EMC | | EMC + 1 wt% FEC | |
|  | Avg. Conc. (%) | SD (%) | Avg. Conc. (%) | SD (%) | Avg. Conc. (%) | SD (%) |
| F1s M–F (LiF) | 0.35 | 0.38 | 1.69 | 1.24 | 1.48 | 0.71 |
| F1s C–F/P–F (LiPF_6_) | 4.41 | 0.68 | 4.15 | 1.23 | 4.39 | 1.17 |
| C–O–C, C–OH | 8.24 | 0.68 | 2.24 | 0.62 | 2.25 | 0.53 |
| N 1s | 2.35 | 0.26 | 0.85 | 0.07 | 0.87 | 0.05 |
| P 2p M-P-X (LiPO_x_F_y_) | 0.25 | 0.03 | 0.62 | 0.36 | 0.36 | 0.06 |
| P 2p P-F (LiPF_6_) | 0.47 | 0.03 | 0.52 | 0.21 | 0.65 | 0.15 |

**Table S3.** Relative atomic concentrations of PVMPO electrodes after 2^nd^ cycle with three different electrolytes.

| Components | Electrolyte (after 2^nd^ cycle) | | | | | |
| --- | --- | --- | --- | --- | --- | --- |
|  | Reference | | EMC | | EMC + 1 wt% FEC | |
|  | Avg. Conc. (%) | SD (%) | Avg. Conc. (%) | SD (%) | Avg. Conc. (%) | SD (%) |
| F1s M–F (LiF) | 0.05 | 0.04 | 1.03 | 0.40 | 1.67 | 0.07 |
| F1s C–F/P–F (LiPF_6_) | 5.30 | 0.5 | 5.82 | 1.09 | 7.75 | 1.01 |
| C–O–C, C–OH | 9.33 | 1.16 | 2.22 | 0.45 | 2.75 | 0.71 |
| N 1s | 2.27 | 0.12 | 1 | 0.08 | 0.93 | 0.03 |
| P 2p M–P–X (LiPO_x_F_y_) | 0.21 | 0.02 | 0.45 | 0.08 | 0.50 | 0.02 |
| P 2p P–F (LiPF_6_) | 0.64 | 0.07 | 0.54 | 0.12 | 0.58 | 0.05 |

**Table S4**. GC-FID analysis of electrolyte composition evolution in reference, EMC, and EMC + 1 wt% FEC systems over 500 cycles.

| Electrolyte | GC-FID | Relative composition (wt%) | | | Error (wt%) |
| --- | --- | --- | --- | --- | --- |
|  | Cycle | EMC | EC | FEC |  |
| Reference | 0 | 68 | 32 | - | 2 |
|  | 200 | 58 | 42 | - | 1 |
|  | 500 | 49 | 51 | - | 2 |
| EMC | 0 | 100 | - | - |  |
|  | 200 | 100 | - | - |  |
|  | 500 | 100 | - | - |  |
| EMC + 1 wt% FEC | 0 | 100 | - | n.d. |  |
|  | 200 | 100 | - | n.d. |  |
|  | 500 | 100 | - | n.d. |  |
